# Early Shift of Attention Is Not Regulated by Mind Wandering in Visual Search

**DOI:** 10.3389/fnins.2020.552637

**Published:** 2020-10-02

**Authors:** Lena Vogelgesang, Christoph Reichert, Hermann Hinrichs, Hans-Jochen Heinze, Stefan Dürschmid

**Affiliations:** ^1^Department of Behavioral Neurology, Leibniz Institute for Neurobiology, Magdeburg, Germany; ^2^Forschungscampus STIMULATE, Otto-von-Guericke University, Magdeburg, Germany; ^3^CBBS – Center of Behavioral Brain Sciences, Otto-von-Guericke University, Magdeburg, Germany; ^4^Department of Neurology, Otto-von-Guericke University, Magdeburg, Germany; ^5^German Center for Neurodegenerative Diseases (DZNE), Magdeburg, Germany

**Keywords:** mind wandering, attentional decoupling, eye tracking, top–down, early attention

## Abstract

Unique to humans is the ability to report subjective awareness of a broad repertoire of external and internal events. Even when asked to focus on external information, the human’s mind repeatedly wanders to task-unrelated thoughts, which limits reading comprehension or the ability to withhold automated manual responses. This led to the attentional decoupling account of mind wandering (MW). However, manual responses are not an ideal parameter to study attentional decoupling, given that during MW, the online adjustment of manual motor responses is impaired. Hence, whether early attentional mechanisms are indeed downregulated during MW or only motor responses being slowed is not clear. In contrast to manual motor responses, eye movements are considered a sensitive proxy of attentional shifts. Using a simple target detection task, we asked subjects to indicate whether a target was presented within a visual search display by pressing a button while we recorded eye movements and unpredictably asked the subjects to rate their actual level of MW. Generally, manual reaction times increased with MW, both in target absent and present trials. But importantly, even in trials with MW, subjects detected earlier a presented than an absent target. The decoupling account would predict more fixations of the target before pressing the button during MW. However, our results did not corroborate this assumption. Most importantly, subject’s time to direct gaze at the target was equally fast in trials with and without MW. Our results corroborate our hypothesis that during MW early, bottom–up driven attentional processes are not decoupled but selectively manual motor responses are slowed.

## Introduction

The human mind wanders frequently away from the conscious perception of external events to focus on the internal milieu (Killingsworth and Gilbert, 2010). Mind wandering (MW) is assumed to bear both a tremendous cognitive resource since it is associated with higher levels of creativity (Baird et al., 2012; Zedelius and Schooler, 2015; Leszczynski et al., 2017) and a harmful impact on life, for instance when thoughts drift away in challenging situations like driving through traffic (He et al., 2011; Kucyi et al., 2013; Yanko and Spalek, 2014). During MW, we are more prone to make errors, especially when withholding an automated button press as in the sustained attention to response task (SART; e.g., Carriere et al., 2008; Smallwood et al., 2008; Seli, 2016; Leszczynski et al., 2017). The attentional decoupling hypothesis predicts that during MW, executive resources shield internally oriented thought flow against perceptual processing of distractors (Christoff et al., 2016) and attention shifts inwards (Smallwood and Schooler, 2006), which was amply attested by reduced EEG response (Smallwood et al., 2008; Kam et al., 2011, 2018).

However, whether MW alters the shifting of attention to external events, as expected by the attentional decoupling hypothesis, or limits online adjustment of motor behavior (Kam et al., 2012; Seli, 2016) is not clear. Importantly, MW (OFF periods) is potentially harmful (He et al., 2011) if reduced sensory processing takes place at the expense of the ability to flexibly shift attention to key features in the environment. Hence, in order to continuously perform an experimental task, humans have to be equipped with the ability to bridge these OFF periods.

We asked the question whether a sustained level of attentional deployment can be found on a behavioral level, which potentially helps to cope with the reduced sensory representation during MW. Manual motor responses alone are not suited to resolve this question because it is unclear whether a delayed reaction is caused by attentional decoupling in early sensory processes or the manual motor response itself is slowed. Eye movements in contrast are generally considered to be a proxy of early attentional orientation, which provides a more direct measure of where attention is deployed (Hoang Duc et al., 2008). Gaze direction can take place without awareness, showing that bottom–up, oculomotor capture by salient stimuli cannot be completely overwritten by top–down influences (Theeuwes et al., 1999). However, whether we can disengage from sensory salience during MW is not clear.

Using the high temporal resolution of eye movement recordings, we tested the influence of MW on eye and manual motor responses. We hypothesize that especially manual responses are affected while bottom–up driven, early eye movements are largely unaffected during MW. We compared the search process using eye movements across the entire display between deep levels of MW (OFF task) and focused attention (ON task) and hypothesized that the time course of eye movements across the visual search display follows the same dynamic in both conditions.

## Materials and Methods

### Participants

Twenty-two participants (mean, 26; *SD*, 5.75; range, 20–43 years, 16 female) with normal vision and without any neurological or psychiatric disorders provided written informed consent prior to the experiment. We included 22 participants since our review of studies on MW and attentional effects as to the number of subjects included revealed that the majority of studies used around 20 subjects. This number of subjects allowed a better comparison with previous studies. All participants received monetary compensation. The Ethical Committee of the Otto-von-Guericke University Magdeburg approved this study.

### Paradigm

**FIGURE 1:**
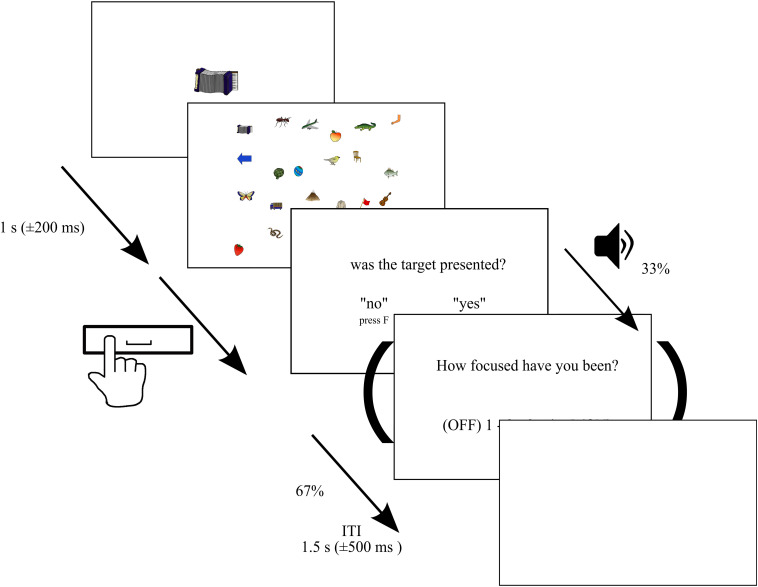
Paradigm. Depiction of the paradigm. Each trial started with the presentation of a target presented at the center of the screen for 1 s (±200 ms) immediately followed by a visual search display consisting of 24 randomly selected objects. In 80% of the trials, the search display contained the target, while in the remaining 20%, the target was not presented. Each of the 250 stimuli served as a target three times across the entire experiment. Subjects were instructed to search the visual display and press the space bar when the search terminated (target found or verified that no target was presented). The display was switched off upon pressing the space bar or remained presented for a maximum of 14 s. Afterward, they were asked to press the “J” key with the right index finger when the target was presented or the “F” button with left index finger when the target was not presented. In 33% of trials, we delivered thought probes initiated by an auditory stimulus asking participants to rate their attentional focus, in the period immediately prior to the probe, on a 5-point scale from 1 (”thoughts were anywhere else”—OFF) to 5 (“thoughts were totally at the task”—ON).

We used a target detection task in a visual search display (see Figure 1). In contrast to previous SART studies, in this paradigm, we asked the subjects to search for a target that changed in each trial. This was used to avoid a high level of fatigue throughout the experiment. The set of stimulus pictures consisted of 250 colored line drawings of objects, animals, or body parts (Rossion and Pourtois, 2004), which differed largely in feature dimension compared to stimuli used in previous MW studies to allow for differences in salience in contrast to previous reading and SART studies. Each trial started with the presentation of one of these objects (*target*) presented at the center of the screen (between approximately 3.4 and 5.2° of visual angle) for a 1,000 ms (±200 ms) immediately followed by the presentation of a visual search display. In each trial, 24 objects were randomly selected and presented on an invisible 4 × 6 grid (1,400 × 900 pixel) with a random shift of ± 40 pixels in vertical and horizontal direction from center points. Within the search display, each stimulus subtended approximately between 1.2 and 2.0° of visual angle. In 80% of the trials, the search display contained the target [target trials (TT)], while in the remaining 20% of trials, the target was not presented [non-target trials (NTT)]. In total, each participant was presented with 750 trials across six blocks. Hence, each stimulus served as a target three times throughout the experiment with an unpredictable order but with the constraint that one and the same object was not the target in subsequent trials. Between blocks, subjects were allowed to rest and initiated the next block on their own. Subjects were instructed to search the visual display and press the space bar when the search terminated (target found or verified that no target was presented). The display was switched off upon pressing the space bar. Trials without pressing the space bar within 14 s were discarded from further analyses. Furthermore, trials with reaction times smaller than 200 ms and exceeding 95% of the distribution of all reaction times were discarded (8% of trials averaged across subjects, *SD*: 4%), too (Samaha and Postle, 2017). After the presentation of the visual search display, the participants were asked to press the “J” key with the right index finger when the target was presented or the “F” button with the left index finger when the target was not presented. The intertrial interval was randomly varied between 1 and 2 s in steps of 100 ms. The experiment was programmed and run with Matlab (R2013a) using the Psychophysics Toolbox extensions (Brainard, 1997; Pelli, 1997) and was presented on a 1,920 × 1,200 pixels hp CN44340CCF monitor at a 60 Hz refresh rate. The stimuli were viewed with a distance of 72 cm from the monitor, and the distance between the eyes and camera was 62 cm in all sessions. The distance was fixed by using a chin rest.

### Experience Sampling

Throughout the experiment, we delivered thought probes in an unpredictable order, asking participants to rate their attentional focus in the period immediately prior to the probe on a 5-point scale from 1 (“thoughts were anywhere else”—OFF) to 5 (“thoughts were totally at the task”—ON). Responses were recorded using the computer keyboard. The probes were pseudo-randomly presented in 33% of the trials after the target absent/present question, such that probes were separated by a minimum of one intervening search trial. The probes were initiated by an auditory stimulus (400 Hz, ca. 60 dB for 200 ms). To increase statistical power, we grouped the five MW ratings in three groups of mental state (OFF, 1 and 2; MID, 3; ON, 4 and 5).

### Eye Movement Recording

For eye movement recording, we used the Eyelink 1000 system operated on Windows 7 and a desktop mounted Eyelink CL camera with a TV lens (35 mm 1:1.6). All participants used a chin and forehead rest with 72 cm distance to the monitor and 62 cm to the camera. In each subject, we tracked the pupil diameter and corneal reflex of the left eye with a sampling rate of 250 Hz. Before each trial block, we performed a calibration session with the built-in 9-point grid method.

### Preprocessing

We used Matlab 2013b (Mathworks, Natick, United States) for all offline data processing. The resulting time series (−1 to 4 s around stimulus onset) were used to characterize eye movement dynamics over the course of visual search. First, we identified trials with low fixation in the baseline period (−1 to stimulus onset). That is, we calculated for each trial the variance of horizontal and vertical eye movements during target presentation. Trials with a variance value above 2 *SD* (indicating low fixation) of all trials were excluded from analyses (11% of trials discarded averaged across subjects, *SD*: 6.9%).

### Data Analysis

Eye tracking data and MW ratings were used for the following analysis steps (all details are explained below). We tested whether MW ratings were correlated to individual mindfulness trait level as assessed by the Mindful Attention and Awareness Scale (MAAS) questionnaire (Brown and Ryan, 2003) (*I—Comparison of mental state and trait mindfulness*). We then assessed for differences in target detection performance between mental states (*II—Comparison of behavioral performance between mental states*). Next, we tested for an increase in reaction times with MW (*III—Increase of manual reaction times as a function of mental state*). In the next step, we quantified the eye movement reaction time (ERT) as the time needed to fixate the target in target trials for the first time and compared differences across mental states with differences of the manual reaction time (MRT) (*IV—eye movement reaction time to the target*), and we tested whether mental states differed in the number of fixations of the target in the visual search display in TT (*V—Comparison of frequency of target fixations*). Finally, we tested whether the search process, spanning fixations, and saccades between stimulus onset and manual motor response differs as a function of mental state (*VI—search routing differences*). All data can be viewed at https://figshare.com/s/81a1ac98f1d3f6b76945.

### Statistical Analysis

To correct statistical significance for multiple comparisons, we compared each statistical parameter against surrogate distributions, which were constructed by randomly yoking labels of the ANOVA or t tests in 1,000 iterations. The comparison against the surrogate distribution has the advantage that test parameters are comparable at all, even if the assumptions of the ANOVA are not met. The violation is likely since manual reaction times always show a skewed distribution with a relatively long tail of longer reaction times and a steep border at shorter reaction times. If the distribution of MRTs can be explained by mental states, then consequently, MRTs grouped according to mental states should show differences in variance. However, in that case, the surrogate distribution is generated under the original sample’s distributional and sphericity properties. Consequently, reported *p*-values represent the statistical significance relatively to the constructed surrogate distribution.

#### I—Comparison of Mental State and Trait Mindfulness

MW and sleepiness frequently co-occur but are distinguishable phenomena with additive effects on task performance (Stawarczyk and D’Argembeau, 2016). We hypothesized that the individual MW rating reflects the individual mindfulness rating as assessed with the MAAS only if MW is less influenced by sleepiness, which in turn is assumed to increase during the experiment. To separate genuine MW from sleepiness in later blocks of the experiment, we correlated MW ratings with individual MAAS scores (average across all ratings in the questionnaire) in two parts of the experiment (*I:* blocks 1–3; *II:* blocks 4–6). To assess significance, Pearson’s correlation coefficients were compared against a surrogate distribution. This surrogate distribution was constructed by randomly reassigning the actual MAAS value of single subjects to MW ratings in 1,000 runs, and the confidence intervals (CI; 99.5%) of a normal distribution were determined.

#### II—Comparison of Behavioral Performance Between Mental States

Separately for the two parts of the experiment, we calculated the performance measure d′ as *z(hits) - z(false alarms)* for each participant and compared performance across the three MW categories using a one-way ANOVA. Significance was determined relative to a permutation-derived surrogate distribution of the interaction effect. The distribution was constructed by randomly reassigning the labels (OFF–MID–ON) in single subjects in 1,000 permutations. This leads to 1,000 surrogate *F*-values. Significance criterion was an *F*-value with *p* < 0.01 within the surrogate distribution of all *F*-values. To anticipate, d′ was relatively high. Therefore, in the following steps, we included only correct trials in the analyses.

#### III—Increase of Manual Reaction Times as a Function of Mental State

We included TT and NTT to assess attentional decoupling during MW. We speculate that if MW indeed leads to attentional decoupling, there should be small differences between MRT in TT vs. NTT in OFF trials, since the target should be missed several times even though we look straight at it. In addition, MRT should show large differences in ON trials because, on average, it takes half the time to correctly report that a target is presented than searching the entire display in NTT (Chen et al., 2011). Hence, an ANOVA with the factors trial type (TT vs. NTT) and mental state (OFF–MID–ON) should reveal an interaction of MRTs between both factors. In contrast, a lack of interaction of trial type and mental state would indicate that MW leads to motor decoupling but not necessarily to attentional decoupling. This ANOVA was done separately for blocks *I* and *II.* Again, significance was determined relative to a permutation-derived surrogate distribution of the interaction effect. The distribution was constructed as outlined above.

#### IV—Eye Movement Reaction Time to the Target

We calculated the ERT, which we defined as the time to fixate the target for the first time within ±40 pixels around the target in the horizontal and vertical direction. We compared ERTs with MRTs in target trials using an ANOVA with the factors motor type (ERT vs. MRT), mental state (OFF–MID–ON), and block. Both ERTs and MRTs were centered to directly compare them as two dependent variables. The attentional decoupling hypothesis would predict that both ERT and MRT are longer in OFF trials compared with ON trials. In contrast, a significant interaction between reaction time type (ERT vs. MRT) and mental state would indicate that eye and manual movements are differently affected by MW. Again, the distribution to assess statistical significance was constructed by randomly reassigning the labels to the single subjects in 1,000 permutations.

#### V—Comparison of Frequency of Target Fixations

We reasoned that the ERT is only meaningful if the number of fixations and time between subsequent fixations does not differ across mental states because subjects could fixate the target but need more subsequent fixations to sample evidence for the presence of the target during MW. Hence, we tested whether mental states differed in the amount of target fixations. The attentional decoupling predicts more fixations prior to the button press during MW likewise assuming that the manual response is preserved. Therefore, differences in reaction time should result from longer search times due to attentional decoupling. Alternatively, if early attentional shifts across the visual search display are unaffected, the number of fixations should not be different between mental states. Differences in manual motor responses should hence result from a specific effect of mind wandering on the decision to execute the manual motor response but not eye movements. We calculated the average number of fixations of the target for TT separately for the three mental states and compared them with a one-way ANOVA with the factor mental state. Then, we calculated the average time between subsequent fixations separately for the three mental states and compared them using a one-way ANOVA with the factor mental state. The surrogate distribution of *F*-values was constructed as outlined above.

#### VI—Search Routing Differences

We tested whether visual search was different between ON and OFF trials. To define gaze direction as a function of time across trials, we calculated a histogram of all vertical and horizontal coordinates at each time point, separately for each subject. This results in high probability values for fixation points before stimulus onset and high probability values for locations on the screen where the stimuli were presented following stimulus onset. These can be identified as colored bands in front of an otherwise dark blue background (locations on the screen where participants did not look at consistently) in Figure 4A. From these probability maps, we extracted three time series defined by gaze to targets on three different distance levels to the center of the visual search display (near–intermediate–far). The resulting probability values of gaze direction were averaged at each time point leading to three time series (near, intermediate, far) for each subject and condition for OFF and ON trials (see Figure 4B). We cross-correlated the time series of each distance across subjects in the first block, resulting in an interindividual similarity measure. These cross-correlations were conducted separately for ON and OFF trials. The strength of the cross-correlation indicates whether search processes differ at the three distance levels between mental states. Further, a time lag between the three distance levels, meant as different amount of time needed for the search process, as indicated by the probability maps, indicates differences between the three distance levels, too. We expect high cross-correlation coefficients (CCC) at each distance level, indicating high similarity across mental states but stronger correlations for targets close to the center of the visual search display where target search starts. We compared CCC between distance levels with a one-way ANOVA with the factor distance level. Furthermore, to investigate whether search processes differed between mental states, we compared CCC and lag coefficients between ON and OFF trials. The lag coefficient (time at which the highest CCC was found) is the direct indicator whether search processes differ in time between mental states. We first compared the lag coefficient across subjects with a one-way ANOVA with the factor distance level. Then, we compared separately for each distance level, whether the distribution of lag coefficients across subjects differed from zero between ON and OFF trials with a one sample t test. Lag coefficient not statistically significantly different from zero indicates that search processes do not differ between mental states.

## Results

### I—Comparison of Mental State and Trait Mindfulness

**FIGURE 2:**
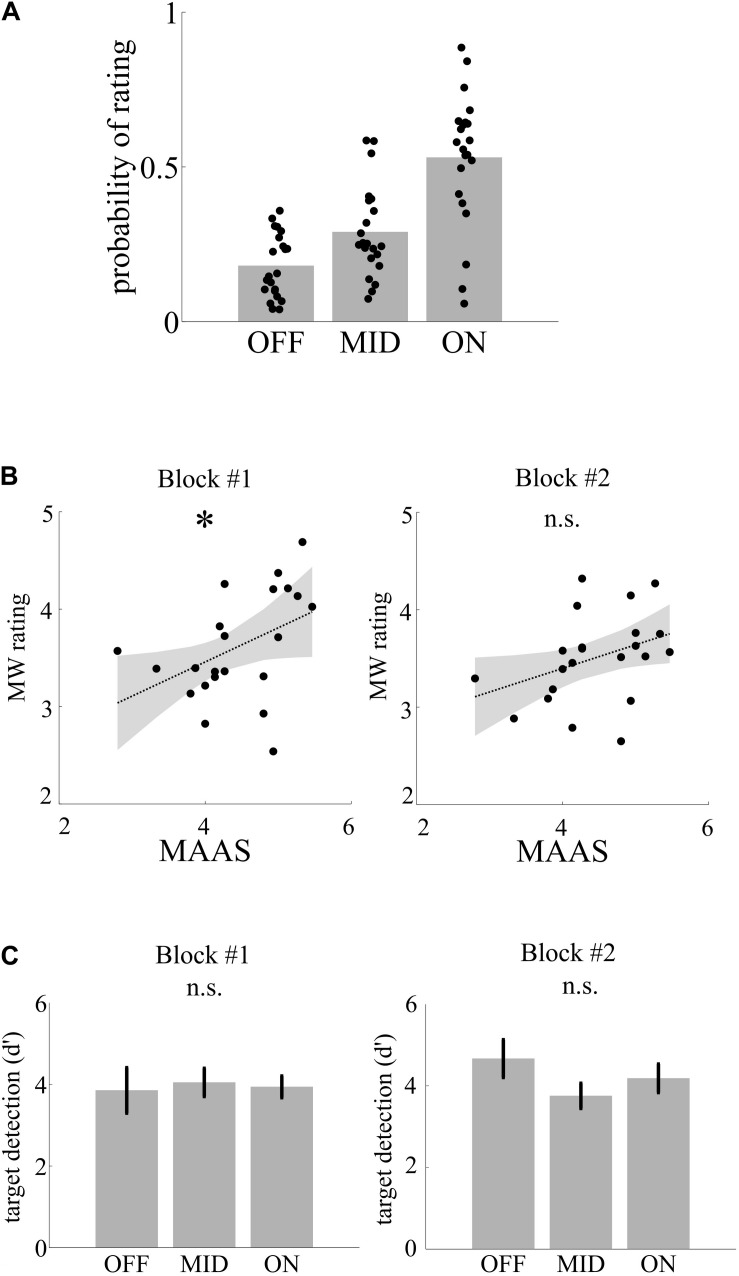
Mental states, trait, and performance. **(A)** Depiction of probability of rating of mental state following thought probes. **(B)** Trait mindfulness as assessed by the Mindful Attention and Awareness Scale (MAAS) is correlated as indicate by the asterisk with the average mind wandering rating following thought probes in the fist but not in the second block. **(C)** Target detection (d′) did not differ with mental state.

To separate genuine MW from sleepiness in later blocks of the experiment, we correlated MW ratings with individual MAAS scores (average across all ratings in the questionnaire) in two parts of the experiment (*I:* blocks 1–3; *II:* blocks 4–6). All of the participants used the full range of mental state ratings in both blocks. Across the experiment, participants used the MW categories differently often [OFF, 17.4%; MID, 27%; ON, 55.6%; *F*_(2, 63)_ = 27.26, *p* < 0.001, η^2^ = 0.47; see Figure 2A]. Individual MW ratings were correlated in the first block (*r* = 0.44, *p* = 0.039; see Figure 2B) but not the second block (*r* = 0.36, *p* = 0.099), indicating that MW ratings in the first part reflects the individual trait mind wandering/mindfulness level, while later in the experiment, individual fatigue additionally affects behavior.

### II—Comparison of Behavioral Performance Between Mental States

We then assessed differences in target detection performance between mental states. Separately for the two parts of the experiment, we calculated the performance measure d′ as *z(hits) - z(false alarms)* for each participant and compared performance across the three MW categories using a one-way ANOVA. Target detection was relatively high (mean across blocks and mental state d′ = 3.94), and there was no significant difference between the MW conditions concerning the target detection rate, neither in the first block of the experiment [*F*_(2_,_63)_ < 1, η^2^ = 0.002] nor in the second [*F*_(2_,_63)_ = 1.26, *p* = 0.310, η^2^ = 0.04; see Figure 2C].

### III—Increase of Manual Reaction Times as a Function of Mental State

**FIGURE 3:**
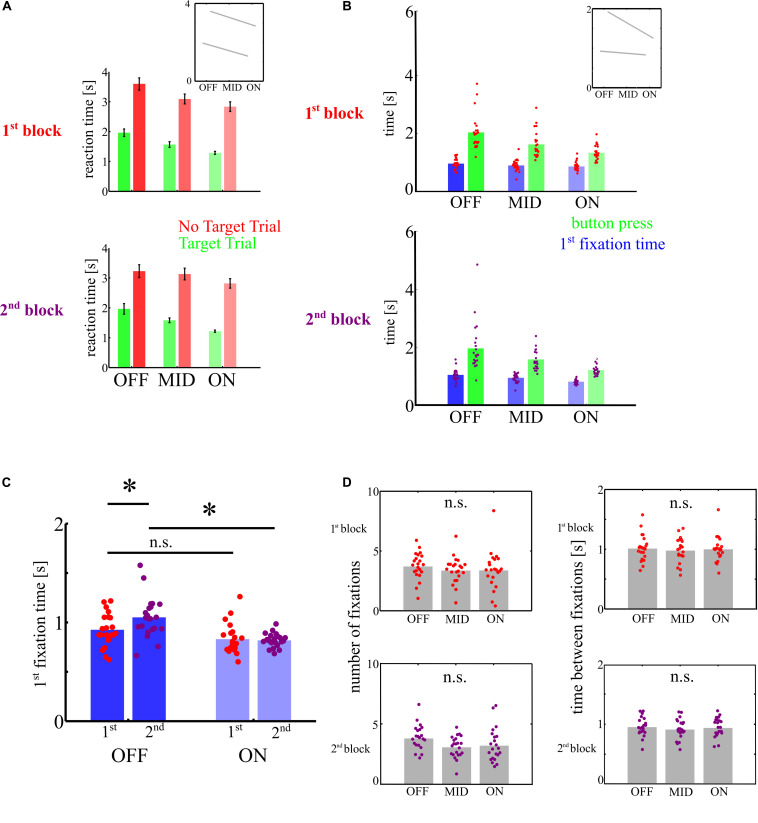
Depiction of reaction times. **(A)** Manual reaction times were longer in non-target trials (NTT) and decreased with attentional focus. However, the difference between target trials (TT) and NTT did not differ with mental state as indicated by the small inset. **(B)** Green bars show manual reaction times (button presses) in TT (as in **A**) together with time until first fixation (blue bars). While manual responses increase with the level of MW, first fixations remain on a constant level as shown in the inset. **(C)** First fixation times were only significantly elevated in OFF trials in the second block, as indicated by the asteriks. **(D)** Neither the number of fixations nor the time between fixations differed between mental states in none of the blocks.

To test for an increase in reaction times with MW, we ran ANOVAs with the factors trial type (TT vs. NTT) and mental state (OFF–MID–ON) separately for blocks *I* and *II*. A lack of interaction of trial type and mental state would indicate that MW leads to motor decoupling but not necessarily to attentional decoupling. We found an increase in MRT with MW and between TT and NTT. All *F*-values were compared against a surrogate distribution (F_*crit*_ = 4.6). We found both longer reaction times in NTT [MRT averaged across mental states: MRT_*NTT*_ = 3.2 s; MRT_*TT*_ = 1.7 s; *F*_(1, 126)_ = 171.40, *p* < 0.001, η^2^ = 0.6] and OFF task trials [MRT averaged across trial types: MRT_*OFF*_ = 2.8 s; MRT_*MID*_ = 2.3 s; MRT_*ON*_ = 2.0 s; *F*_(1, 126)_ = 10.70, *p* < 0.001, η^2^ = 0.2] but no interaction between both factors [*F*_(1, 126)_ = 0.08, *p* = 0.740; see Figure 3A] in the first block. Similarly, we found a main effect of trial type [MRT_*NTT*_ = 3.1 s; MRT_*TT*_ = 1.6 s; *F*_(1, 126)_ = 135.9, *p* < 0.001, η^2^ = 0.5] and MW [MRT_*OFF*_ = 2.5 s; MRT_*MID*_ = 2.3 s; MRT_*ON*_ = 2.0 s; *F*_(1, 126)_ = 6.2, *p* < 0.001, η^2^ = 0.1] and also no interaction effect in the second block [*F*_(1, 126)_ = 0.8, *p* = 0.561; see Figure 3A].

### IV—Eye Movement Reaction Time to the Target

We compared ERTs with MRTs in target trials using an ANOVA with the factors motor type (ERT vs. MRT), mental state (OFF–MID–ON), and block. When we compared the centered MRTs and ERTs across blocks, we found a main effect of MW [*F*_(2, 252)_ = 30.97; *p* < 0.001]. Importantly, we found an interaction effect between both factors [*F*_(2, 126)_ = 12.16, *p* < 0.001]. We did not find a main effect of block. When we compared MRTs and ERT separately for the two blocks, we found for MRT in the first block a main effect of MW [*F*_(2, 126)_ = 14.18; *p* < 0.001, η^2^ = 0.19, Kruskal–Wallis test: χ^2^ = 21.7; *p* = 1.8 × 10^–5^]. Most importantly, we found, in addition, a highly significant interaction [*F*_(2, 126)_ = 8.03, *p* < 0.0001, η^2^ = 0.12] between both factors (see Figure 3B). In the second block, we found a main effect of MW for MRT [*F*_(2, 126)_ = 16.91, *p* < 0.001, η^2^ = 0.21, Kruskal–Wallis test: χ^2^ = 26.5; *p* = 1.7 × 10^–6^] but only a trend of interaction [*F*_(2, 126)_ = 4.71, F_*crit*_ = 4.6, η^2^ = 0.06; see Figure 3B]. We tested whether ERT differed between first and second block. We found a significant interaction between the factors MW and block [*F*_(2, 126)_ = 4.35; *p* = 0.006, η^2^ = 0.1; see Figure 3C]. In planned comparisons, we found that ERT differed between ON and OFF in the second block (*t*_*crit*_ = 2.4, *t*_21_ = 5.82; *p* < 0.001, Kruskal–Wallis test: χ^2^ = 23.4; *p* = 8.1 × 10^–6^) and in OFF trials between first and second block (*t*_21_ = 2.56; *p* = 0.007). However, we did not find such difference between ON and OFF trials in the first block (*t*_21_ = 2.10; *p* = 0.12, Kruskal–Wallis test: χ^2^ = 5; *p* = 0.091; see Figure 3C). Hence, in contrast to attentional lapses due to the individual fatigue level MW, associated with the trait level of mindfulness, does not impact eye movements. Note that the trend of interaction indicates that ERT is less influenced by MW than MRT.

### V—Comparison of Frequency of Target Fixations

We reasoned that the ERT is only meaningful if the number of fixations and time between subsequent fixations does not differ across mental states because subjects could fixate the target but need more subsequent fixations to sample evidence for the presence of the target during MW. Hence, we tested whether mental states differed in the amount of target fixations. The number of target fixations did not differ between mental states, neither in the first block [mean OFF, 3.7; MID, 3.4; ON, 3.4; SD OFF, 1.1; MID, 1.2; ON, 1.7; *F*_(1, 126)_ < 1] nor the second block [mean OFF, 3.8; MID, 3.1; ON, 3.3; SD OFF, 1.1; MID, 0.9; ON, 1.4; *F*_(1, 126)_ = 2.51; *p* = 0.099; see Figure 3D]. In addition, the average time needed between consecutive fixations did not differ between mental states, neither in the first block [mean OFF, 1.0; MID, 0.9; ON, 1.0; SD OFF, 0.21; MID, 0.2, ON, 0.21; *F*_(1, 126)_ < 1] nor the second block [mean OFF, 0.96; MID, 0.92; ON, 0.95; SD OFF, 0.15; MID, 0.17; ON, 0.16; *F*_(1, 126)_ < 1].

### VI—Search Routing Differences

**FIGURE 4:**
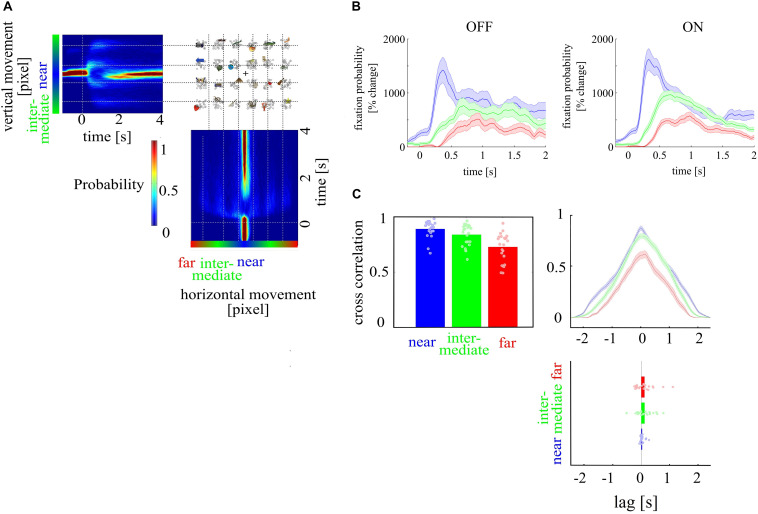
Depiction of search routing. **(A)** Probability values for fixation points over time for horizontal and vertical eye movements, outlining high fixations before stimulus onset and low fixations after stimulus onset. **(B)** shows the gaze direction as a function of time. The gaze direction is shown as the probability to fixate targets at the three different distance levels in percent change over baseline period for OFF and ON trials. **(C)** Cross-correlation of time series for OFF and ON trials for the three target distance levels [near (red), intermediate (green), far (blue)] showed that gaze direction to targets at the different distance levels were highly correlated. The time lag coefficient, an indicator whether search processes differ in time between mental states, showed no difference across distance levels. Gaze direction to targets at near, intermediate, and far distance level were highly correlated between ON and OFF trials and differed in correlation strength. Time lag coefficients did not differ across distance level, and none of the distance levels was different from zero lag.

We tested whether visual search was different between ON and OFF trials. We compared CCC between distance levels with a one-way ANOVA with the factor distance level. Furthermore, to investigate whether search processes differed between mental states, we compared CCC and lag coefficients between ON and OFF trials. Then, we compared separately for each distance level whether the distribution of lag coefficients across subjects differed from zero between ON and OFF trials with a one sample t test. Lag coefficient not statistically significantly different from zero indicate that search processes do not differ between mental states. We found that gaze directions to targets at a near, intermediate, and far distance level were highly correlated (near, 0.89; intermediate, 0.84; far, 0.73) between mental states (ON and OFF) and differed in correlation strength [*F*_(1, 126)_ = 14.31; *p* < 0.001]. However, lag coefficients did not differ across distance levels, and at none of the distance levels’ cross-correlation lag was different from zero lag (near: 0.03, *p* = 0.098; intermediate: 0.11, *p* = 0.094; far: 0.11, *p* = 0.121, see Figure 4C).

## Discussion

MW describes the alternating gain of consciousness of the external and internal milieu. The attentional decoupling hypothesis propagated the view that while we focus on the internal milieu, sensory processing of the environment is decoupled, since manual responses are slowed or could not be withhold during MW. However, whether MW alters early sensory processing and prevents shifting attention to external events, like expected under the attentional decoupling hypothesis, or limits later processes, like the online adjustment of motor behavior (Kam et al., 2012; Seli, 2016), was not experimentally tested so far. In the current study, we tested the influence of MW on eye movements and manual motor responses, hypothesizing that especially manual responses are affected while early eye movements are largely unaffected during MW. We found that MW slows manual response, both when visual search displays contained the target or not and that, in contrast to manual responses, oculomotor responses to targets did not differ between MW and no MW but were affected in later trials when performance might be influenced by fatigue. Furthermore, the search process itself, where attention is routed across the stimuli, is not affected by MW, like expected. In conclusion, these results confirm our presumption that the top–down influence of MW does not decouple early oculomotor processes, as emphasized by the attentional decoupling hypothesis.

Our results indicate that manual motor responses are generally slowed during MW. While potential benefits of MW are rarely covered in previous research, its negative effects determined the view on MW. Reading studies showed that MW drastically reduced reading comprehension (Schooler et al., 2004), leading to the attentional decoupling hypothesis. Furthermore, behavioral motor performance was systematically altered during MW in previous studies on the SART. Manual responses reliably shift to a more automatic and/or degraded state (Weissman et al., 2006; Carriere et al., 2008; Smallwood et al., 2008; Reichle et al., 2010), such that reaction times (RTs) decrease and error rates increase compared to ON-task states (Smallwood et al., 2004; Franklin et al., 2011). Hence, manual motor responses lack in flexibility during mental states of MW. In a corresponding manner, control and adjustment of motor behavior in a motor tracking task is reduced during MW (Kam et al., 2012). These studies are partly in line with our results. The more participants are OFF task, the longer are manual reaction times, indicating less flexibility. However, trials with and without targets usually require self-terminating and exhaustive processes, respectively (Van Zandt and Townsend, 1993). This is an important contrast to assess attentional decoupling during MW. If MW indeed leads to full attentional decoupling (under the premise that manual motor responses are unaffected), there should be no differences between manual reaction times in TT vs. NTT during MW. This effect occurs since the target should be missed several times even though we look straight at it. In contrast, reaction times should be different when subjects report to be ON task because, theoretically, on average, it takes half the time to correctly report the target in TT than searching the entire display in NTT (Chen et al., 2011). However, we did not find an interaction between ON and OFF task trials in terms of reaction time differences between TT and NTT. Instead, reaction times were smaller for TT than for NTT during MW even though generally elevated in OFF task trials. Furthermore, they correctly terminated the search process in TT even though they subjectively rated not paying attention to the task. This would rather be evidence for generally slowed motor response.

However, motor slowing, which we found in our study, might in part explain behavioral errors in previous studies. MW manifests behaviorally, especially in highly automated tasks like reading or the SART. We and a previous study (Kam et al., 2012) found a selective impact of MW on the manual motor component. We speculate that the inhibition of manual motor responses is beneficial since it allows to prevent from overhasty decisions. This general slowing would also explain the behavioral decrements that were observed in SART experiments. We hypothesize that cortical areas responsible for the initiation of manual motor responses are suppressed during MW (OFF task trials). MW and focused attention are associated with activity of the default mode network (DMN; Raichle et al., 2007; Mittner et al., 2014, 2016; Kucyi et al., 2016; Zhou and Lei, 2018) and the dorsal attention network (Fox et al., 2016; Kucyi et al., 2016), respectively, while it is assumed that there is a stronger motor preparation in ON task trials (Sormaz et al., 2018). This is in line with studies showing that faster reaction times are associated with activity in the supplementary motor area (SMA) and slower reaction times are associated with activity in the default mode network, which identifies their different roles in vigilance (Hinds et al., 2013). Hence, we hypothesize a selective control effect of the DMN on SMA presumably to prevent overhasty decisions during MW.

Performance reduction during MW could also be explained by boredom or fatigue due to low task engagement, which we separated from MW using the trait MAAS, a 15-item scale designed to assess a core characteristic of mindfulness. The MAAS is highly correlated with performance failures due to failures of sustained attention especially reaction times in the SART and is assumed to directly reflect lapses of attention that lead to errors (Cheyne et al., 2006). The MAAS correlated only in the first part of our study, indicating that at least in the first part, we indeed tested MW as studied with SART, but additive effects of MW and sleepiness mutually influenced performance (Stawarczyk and D’Argembeau, 2016). In contrast to previous studies, we did not find difference in signal detection, corroborating our claim that attention is not decoupled in OFF trials when task engagement is necessarily high.

In contrast to previous studies, we used a paradigm with highly salient target, which changed from trial to trial. Previous studies on MW that focus on reading or the SART typically rely on highly automated performance. This is important since salience is typically reduced in reading tasks compared to the stimuli used in our study. Despite differences in the cognitive requirements, the monotonic nature of our experiment induced MW similarly. Subjects in our study reported MW as often as the average OFF reports in previous reports (Schooler et al., 2004; Reichle et al., 2010; Smilek et al., 2010; He et al., 2011) and generally more ON than OFF trials (Ward et al., 2013). Hence, differences in the number of OFF periods cannot explain differences in reaction times. Despite the differences in our task compared to previous studies, we found that subjective reports revealed different levels of MW (Schad et al., 2012) reflected by an increasing sluggishness of manual motor responses from ON to OFF task trials. Schad et al. (2012) proposed that attentional decoupling during MW is rather dimensional than dichotomous. According to this view, low-level processing might be gradually intact, and only high-level processing is decoupled during MW. Complementing this hierarchical hypothesis, we found that processes at the lowest level of attentional processing are unaffected predominantly; manual motor responses are affected by MW.

MW studies generally bear the disadvantage that participants have to indicate that they experience MW. Self-caught procedures require meta-awareness of MW, while probe-caught procedures, as used in our study, circumvent this problem. One disadvantage of using probe-caught procedures is that only a fraction of trials can be used to study physiological indicators of MW. The search for reliable, objective indications of MW is therefore at the heart of the field. Eye movements (He et al., 2011; Albert et al., 2018; Steindorf and Rummel, 2019) or pupil size (Hartmann et al., 2015; Unsworth and Robison, 2016; Huijser et al., 2018; Konishi et al., 2017) are potential candidates, since gaze parameters provide real-time indices of the information processing priorities of the visual system (Krasich et al., 2018). In contrast to other studies, we found no differences in dwell times during MW (Krasich et al., 2018) and fixation times (Reichle et al., 2010) with MW. Task engagement could explain differences in terms of eye movement parameters with previous studies in which dwell times are usually elevated during MW, since in our visual search paradigm stimuli differed in salience and task engagement from previous studies. As mentioned above, this is important since salience is typically reduced in reading tasks, and a low cognitive demand paired with a high level of attention only eventually pronounces states of MW (He et al., 2011). He et al. (2011) have found an interaction of horizontal eye movements (but not vertical) between the mental state and the experimental conditions, varying in cognitive demand. However, it is not clear from their study whether horizontal eye movements differed in their high cognitive load condition when attention is high between ON and OFF task mental states. Hence, when salience is high, physiological differences between ON and OFF task states might shrink particularly on the level of eye movements. Eye movements, in contrast to manual response, are less variable across mental states, indicating that target selection is unaffected by MW. The shift of attention and saccades implemented in the visual system work jointly to allow the selection of objects, features, and locations with the greatest momentary need. Eye movements are mainly driven by neurons in the frontal eye field (FEF), superior colliculi (SC), and lateral intraparietal cortex (LIP), where neurons select targets independently of eye movements initiation (Horwitz and Newsome, 1999; Murthy et al., 2001). Presaccadic shifts of attention enhance processing at the target goal location to mediate changes of the strength of perceptual representations, select targets for encoding in memory, exclude noise, or change the level of internal noise (Zhao et al., 2012). The integration of all these information starts already with stimulus onset (Caspi et al., 2004). This saccadic shift can be initiated even without awareness (Hoang Duc et al., 2008) and is purportedly driven by salience (Parkhurst et al., 2002) so that we assume that early visual attention, as reflected by eye movements, seems to be impenetrable to MW. This explains why we react slower but appropriately to challenging situations during traffic, despite MW (He et al., 2011; Yanko and Spalek, 2014).

## Conclusion

Our results imply that the early process of executing saccades to fixate the target is not affected by MW. This is supported by a growing body of evidence indicating less top–down influences on initial processing steps as previously assumed by the advent of predictive coding accounts (Rao and Ballard, 1999) tested with functional MRI (fMRI). Especially the first forward sweep of attention is assumed to be completely stimulus driven, and the earliest top–down effects on the earliest visual cortical processing takes place only after 80 ms poststimulus (Theeuwes, 2010). Without any doubt, bottom–up processes are modulated top–down; however, these studies and our findings indicate that initial visual afferent activity may be impenetrable to top–down influence like MW. This relates to the search process in general in which ensuing saccades are guided mostly by information presented before the first saccade rather than information presented during the intersaccadic interval (Caspi et al., 2004). Scanning the visual environment can be regarded as a continuous and not a stepwise process as sequences of fixations might imply. In sum, our results show the necessity of more research into attentional decoupling independent of sensory decoupling and motor behavior to better understand the impact of mind wandering on the various stages of attentional allocation, perception, and action selection.

## Data Availability Statement

The raw data supporting the conclusions of this article will be made available by the authors, without undue reservation, to any qualified researcher.

## Ethics Statement

The studies involving human participants were reviewed and approved by the Ethical Committee of the Otto-von-Guericke University Magdeburg. The patients/participants provided their written informed consent to participate in this study.

## Author Contributions

LV and SD conceived and designed the experiment and collected the eye tracking data. CR and SD analyzed the data. LV, SD, CR, HH, H-JH, and SD interpreted the data. SD and LV wrote the manuscript. All authors contributed to the article and approved the submitted version.

## Conflict of Interest

The authors declare that the research was conducted in the absence of any commercial or financial relationships that could be construed as a potential conflict of interest.
